# Climate change and African trypanosomiasis vector populations in Zimbabwe's Zambezi Valley: A mathematical modelling study

**DOI:** 10.1371/journal.pmed.1002675

**Published:** 2018-10-22

**Authors:** Jennifer S. Lord, John W. Hargrove, Stephen J. Torr, Glyn A. Vale

**Affiliations:** 1 Department of Vector Biology, Liverpool School of Tropical Medicine, Liverpool, United Kingdom; 2 SACEMA, University of Stellenbosch, Stellenbosch, South Africa; 3 Natural Resources Institute, University of Greenwich, Chatham, United Kingdom; Africa Program, UNITED STATES

## Abstract

**Background:**

Quantifying the effects of climate change on the entomological and epidemiological components of vector-borne diseases is an essential part of climate change research, but evidence for such effects remains scant, and predictions rely largely on extrapolation of statistical correlations. We aimed to develop a mechanistic model to test whether recent increases in temperature in the Mana Pools National Park of the Zambezi Valley of Zimbabwe could account for the simultaneous decline of tsetse flies, the vectors of human and animal trypanosomiasis.

**Methods and findings:**

The model we developed incorporates the effects of temperature on mortality, larviposition, and emergence rates and is fitted to a 27-year time series of tsetse caught from cattle. These catches declined from an average of c. 50 flies per animal per afternoon in 1990 to c. 0.1 in 2017. Since 1975, mean daily temperatures have risen by c. 0.9°C and temperatures in the hottest month of November by c. 2°C. Although our model provided a good fit to the data, it cannot predict whether or when extinction will occur.

**Conclusions:**

The model suggests that the increase in temperature may explain the observed collapse in tsetse abundance and provides a first step in linking temperature to trypanosomiasis risk. If the effect at Mana Pools extends across the whole of the Zambezi Valley, then transmission of trypanosomes is likely to have been greatly reduced in this warm low-lying region. Conversely, rising temperatures may have made some higher, cooler, parts of Zimbabwe more suitable for tsetse and led to the emergence of new disease foci.

## Abstract 2

Jennifer Lord and colleagues model how climate change will affect Tsetse fly numbers in the Zambezi Valley in Zimbabwe; falling populations may impact cases of human and animal trypanosomiasis.

## Author summary

**Why was this study done?:**

- Tsetse flies are blood-feeding insects that transmit pathogens causing fatal diseases of humans and livestock across sub-Saharan Africa.
- The birth and death rates of tsetse are influenced by environmental conditions, particularly temperature.
- Since 1975, mean daily temperatures at Rekomitjie, a research station in the Zambezi Valley of Zimbabwe, have risen by c. 0.9°C and temperatures in the hottest month of November by c. 2°C. These increases in temperature may have impacted tsetse populations and the diseases they transmit.

**What did the researchers do and find?:**

- Since the 1960s, wild tsetse have been caught regularly from cattle for insecticide tests conducted at Rekomitjie.
- Prior to 1990, catches were on average >50 flies per animal per afternoon; however, in 2017, teams frequently failed to catch a single fly.
- A mathematical model of tsetse population change, which included temperature-dependent rates for births and deaths, suggests that the decline in tsetse is related to rising temperatures.

**What do these findings mean?:**

- Our findings provide a first step in linking the effects of increasing temperatures to the distribution of diseases caused by tsetse.
- If the effect extends across the Zambezi Valley, then tsetse-borne disease is likely to have been reduced across the region. Conversely, rising temperatures may have made some higher, cooler areas more suitable, leading to the emergence of new disease foci.

## Introduction

Tsetse flies (*Glossina* spp.) transmit protozoa of the genus *Trypanosoma* that cause sleeping sickness—human African trypanosomiasis (HAT)—in humans. The initial phase of HAT is characterised by intermittent fever and joint pains; thereafter, there are sleeping difficulties and confusion. Without treatment, the disease is fatal. Parasites of this genus also cause nagana—animal African trypanosomiasis (AAT)—in livestock.

In 2015, HAT was responsible for c. 202,000 Disability-Adjusted Life Years (DALYs) [1]. The most recent global estimates indicate that AAT kills approximately 1 million cattle per year [2], with approximately 55 million cattle at risk [3].

In addition to the DALYs resulting from HAT, AAT also has substantial impacts on human health by reducing the supply of meat and milk, as well as animal draft power for crop production. These losses affect not only human nutrition but also the agricultural incomes that allow access to education and healthcare [4]. A study in 1999 indicated that the annual economic losses from meat and milk production alone were c. US$1 billion at current prices [5].

In Africa, there has been an increase in temperature of c. 1.5°C between 1900 and the 1990s [6]. However, the effects of recent and future climate changes on the distribution of tsetse and other vectors, and their associated diseases, remain poorly understood [7,8]. There is disagreement, for example, about whether the resurgence of malaria in the East African highlands in the 1990s was caused by rising temperatures or by increasing levels of drug resistance and decreasing control efforts [9–13]. Resolution of the debate is made more complex by the apparent absence of data on changes in vector population levels and biting rates.

Increases in global temperatures since the late 1800s [14] have led to shifts in the ranges of many animals [15]. Insects in particular are sensitive to changes in temperature, with consequences for the transmission of vector-borne pathogens [7,16,17]. Mechanistic models, capable of explaining how recent climate change [14] has affected vector distribution and abundance, could be used to predict future disease risks [16], but existing studies often rely instead on statistical correlations [8,18–20].

In general, the ways in which climate change will affect infectious disease burden in sub-Saharan Africa is poorly understood because of a lack of empirical evidence [21]. It has been suggested that requirements for accepting a ‘causal’ relationship between climate change and changes in human health outcomes for vector-borne diseases should, as a minimum, include (i) evidence of biological sensitivity to climate, (ii) meteorological evidence of climate change, and (iii) evidence of entomological and/or epidemiological change in association with climate change [8].

For vector-borne diseases, the difficulty is the ability to separate climatic effects from those originating from other environmental, ecological, and sociological changes influencing the population dynamics of parasites and vectors. Contributing to this difficulty is a lack of contiguous data on vector abundance and detailed records of local climate. Work on tsetse and trypanosomiasis carried out at Rekomitjie Research Station in the Mana Pools National Park, Zimbabwe over the last 59 years provides a valuable exception to this rule, producing long-term datasets for both vector abundance and climate profile.

The study site is located >10 km inside a protected area (S1 Fig). According to the World Database on Protected Areas (https://protectedplanet.net/), the Mana Pools National Park, together with its adjoining Sapi and Chewore Safari Areas and the adjacent Hurungwe Safari Area, has a total area of 9,660 km^2^. It has been free of agricultural settlement since 1958, when the people living there were relocated [22]. Since then, the combined area has been protected against settlement, agriculture, and illegal hunting and logging and was designated a UNESCO World Heritage Site in 1984. In this area, HAT occurs, and tsetse populations have not been exposed to any form of control. In addition, being situated in a protected area, the region has not been subject to other deliberate environmental or sociological change. Analyses by Hansen and colleagues [23] show that this area experienced <0.5% woodland loss between 2000 and 2010, with the majority of the 30 m × 30 m pixels in the Hansen and colleagues dataset within Mana Pools consisting of at least 10% wooded cover (S1 Fig). In addition, an aerial survey for elephant and buffalo in 2014 [24] indicated that, in the 200 km^2^ around Rekomitjie, there was an average of 1.6 elephants and 7.3 buffalo per km^2^. Vale and colleagues [25] showed that c. 2 elephants per km^2^ can provide about half of the diet of savanna species of tsetse and can support a population of flies even when alternative hosts are heavily depleted.

The data available therefore provide the possibility of developing temperature-driven models for tsetse population dynamics. Such models could be used to predict the present and future distribution of tsetse in Africa. Given that there is never any cyclically transmitted African trypanosomiasis without the presence of tsetse, such models will provide a more powerful approach for estimating changes in the distribution of human and animal trypanosomiases.

Tsetse flies are poikilotherms, and their development and mortality rates are dependent on temperature [26–30]. We aimed to use data on temperature and tsetse abundance at Rekomitjie to test whether observed increases in temperature over recent years are sufficient to explain the observed decline in the local tsetse population since the 1990s. To do this, we used a mechanistic model of tsetse population dynamics that incorporates the effect of temperature on adult and pupal mortality and rates of larval deposition and pupal development, established from laboratory and field studies [26–30]. We fitted the model to a 27-year dataset of *Glossina pallidipes* numbers caught from bait oxen.

## Methods

The methods for the production of data for tsetse and climate were not guided by an analysis plan for the present study. Instead, the climate data were produced as a standard procedure at the research station over the past 59 years, and the tsetse data were obtained from previous studies [31–36].

### Temperature data and analysis

Daily records of rainfall and minimum and maximum temperature have been kept at Rekomitjie since 1959. Staff at Rekomitjie, operating in accord with directions from the Zimbabwe Department of Meteorological Services, made recordings at 7:00 AM each day from maximum and minimum mercury thermometers housed in a Stevenson screen. The location of the screen is at 16° 10′S 29° 25′E, altitude 503 m. To quantify changes in the mean temperature over time, we first calculated mean monthly temperatures between October 1959 and June 2017. Then, using a reference period between January 1960 and December 1989, we calculated monthly temperature anomalies by subtracting the reference mean from the actual mean. We smoothed the temperature anomaly data using a 5-year running mean, as done for previous analyses of regional and global changes in temperature [37–39]. In addition, a time series linear regression model was fitted to the mean monthly temperature data using the ‘tslm’ function from the forecast package [40]—a wrapper for fitting linear models allowing for a trend variable. We subsequently employed the fitted trend to estimate the change in monthly temperature, from the peak in 1975 to the peak in 2017, and the 95% prediction intervals, using the forecast function in R [41].

### Tsetse data

Sampling of tsetse at Rekomitjie, in pursuit of various ecological and behavioural studies, has suggested a decline in tsetse abundance in the last two decades. It is difficult to interpret the catches confidently because they have been made using widely different methods at irregular intervals. From 1966, however, fed female *G*. *pallidipes* have been collected from stationary oxen at Rekomitjie, with the sole original aim of providing test insects for bioassays [31–36]. Because these collections were made using a single sampling system, run at approximately the same time each day, the change in the numbers collected offer an indication of the extent of the decline in tsetse abundance over recent decades.

Catches were made for 3 hours in the afternoon during the period of peak tsetse activity [42]. Each collection team comprised two hand net catchers and an ox, operating within 2 km of the research station. Each team operated at least 200 m from other teams, in areas chosen to maximise catches in accord with seasonal changes in the distribution of tsetse between vegetation types [43]. In the 1960s, it was usual for each team to take enough tubes to collect a maximum of about 50 flies each day. This quota was set in consideration of the minimum expected catch at that time and has been maintained at this level ever since, even though it has proved impossible to meet the quota in the last two decades. Daily records are available from 1990 for the number of catching teams employed, and for the catch of each team. The monthly averages of the number of flies caught per team per day are taken as indices of fly abundance. Prior to 1990, tsetse catches regularly reached the upper limit of 50 flies; thereafter, this hardly ever occurred. Fitting the model only to catch data for the period after 1990 ensured that there was no truncation of data used in the fitting procedure.

### Modelling tsetse population dynamics

Tsetse females give birth, approximately every 7 to 12 days [28], to a single larva, which immediately burrows into the ground and pupates, emerging 30 to 50 days later as a full-sized adult [44]. Female adult flies can live for up to 200 days [45]. As quantified by researchers in the laboratory and field, larviposition and pupal emergence rates are dependent on temperature, as are the mortality rates of both pupae and adults [26–28,30]. Preliminary analyses suggested that the inclusion of temperature-dependent mortality rates was sufficient to model the observed decline. In response to suggestions from peer reviewers, we also reanalysed the data using models that included functions for temperature-dependent larviposition and pupal emergence rates.

Hargrove [29,30], using data from mark-recapture experiments on Antelope Island, Lake Kariba, Zimbabwe, showed that for *G*. *pallidipes*, female adult mortality increases with temperatures above 25°C. In our ordinary differential equation (ODE) model of tsetse population dynamics, described below, we therefore model female adult losses per day (*μ*_*A*_) due to temperate-dependent mortality using

$$\mu_{A}=\left\{ \begin{array}{l} a_{1}\;T\leq 25\\ a_{1}\;\exp\left(a_{2}(T-T_{1})\right)\;T>25 \end{array}\right.$$ (1)

where *T* is temperature in°C. *T*_*1*_ is not a parameter but is a constant set to 25 to ensure that *a*_*2*_ is in a convenient range.

For pupae, the relationship between mortality rate per day (*μ*_*p*_) and temperature was quantified by Phelps [27] in the laboratory. The data from these experiments show that pupal survival to adulthood is highest for temperatures between about 20°C and 30°C. As temperatures depart from this range, the mortality rises sharply, resulting in a U-shaped curve. This form of relationship has also been documented for various other insects [46] and, for tsetse, can be suitably modelled using the sum of two exponentials:

$$\mu_{p}={b_{1}+b}_{2}\;\exp\left(-b_{3}(T-T_{2}\right)+b_{4}\;\exp\left(b_{5}(T-T_{3})\right)$$ (2)

where *T* is temperature in°C. *T*_*2*_ and *T*_*3*_ are not parameters but are constants chosen to ensure that the coefficients *b*_*3*_ and *b*_5_ are in a convenient range and were set to 16°C and 32°C, respectively.

Phelps also quantified the daily rate of pupal development (*β*) in *G*. *m*. *morsitans* as a function of constant temperature in the laboratory, fitting the data using the function [29]

$$\beta =c_{1}/(1+\exp\left(c_{2}+c_{3}T\right)$$ (3)

where, for females, the fitted estimates were *c*_*1*_ = 0.05884, *c*_*2*_ = 4.8829, and *c*_*3*_ = −0.2159.

The effects of temperature on pupal development and mortality rates in the laboratory are supported by work showing similar effects in the field [44,47–49].

Lastly, using ovarian dissection data from marked and released *G*. *m*. *morsitans* and *G*. *pallidipes* at Rekomitjie, Hargrove [28] showed that the larviposition rate per day (*ρ*) increases linearly between 20°C and 30°C. We therefore assume a linear increase in larviposition rate with temperature using the equation

$$\rho =d_{1}+d_{2}(T-T_{4})$$ (4)

where *T*_*4*_ was set to 24°C. The time taken for a female tsetse to produce her first larva is longer than for subsequent larvae. Accordingly, the values for *d*_*1*_ and *d*_2_ in Eq 4 are lower for nulliparous females (*d*_*1*_ = 0.061 and *d*_*2*_ = 0.002 [*ρ*_*n*_]) than for parous females (*d*_*1*_ = 0.1046 and *d*_*2*_ = 0.0052 [*ρ*_*p*_]) [30].

Considering the above temperature-dependent processes, and using the outlined functions for the five parameters *μ*_*A*_, *μ*_*P*_, *β*, *ρ*_n_, and *ρ*_p_, we model changes in the numbers of *G*. *pallidipes* female adults (*A*) and pupae (*P*) using three ODEs:

$$\frac{dP}{dt}=\rho_{n}A_{n}+\rho_{p}A_{p}-(\beta +\mu_{P}+\delta P)P$$ (5)

$$\frac{dA_{n}}{dt}=\frac{\beta }{2}P-(\mu_{A}+\rho_{n})A_{n}$$ (6)

$$\frac{dA_{p}}{dt}=\rho_{n}A_{n}-\mu_{A}A_{p}$$ (7)

Pupae are produced by nulliparous (*A*_*n*_) and parous (*A*_*p*_) adult females at rates *ρ*_*n*_ and *ρ*_*p*_, respectively. Losses from the pupal stage are due to pupae emerging as nulliparous adults (*A*_*n*_), at rate *β*/2, to density-dependent mortality, with coefficient *δ* and mortality *μ*_*P*_. Losses from the nulliparous adult stage are due to first larviposition at rate *ρ*_*n*_ and mortality (*μ*_*A*_), assumed equal for both nulliparous and parous females.

### Model fitting

As initial starting estimates for the parameters in the model described in Eqs 5–7, we used the published [26,28,30] fitted values for larviposition and pupal emergence rates as described above (Eqs 3 and 4). For adult and pupal mortality, we fitted the functions in Eqs 1 and 2 to published data—described above and elsewhere [27,29,30]—using nonlinear least squares regression.

It was not necessary to vary all parameters in the ODE model to get a reasonable fit to the bioassay catch data. The only parameter in the population dynamic model for which we did not have an initial starting estimate from published data was the density-dependent mortality coefficient (*δ*). For model fitting, therefore, we first allowed only this parameter to vary while keeping all other parameter values fixed. We then fitted the model to the average monthly tsetse catches allowing just *δ* and the parameters for adult mortality (*a*_*1*_ and *a*_*2*_) to vary, followed by those for just pupal mortality (*b*_*1*_ to *b*_*5*_) and lastly for *δ* and both mortality functions. For pupal mortality, it was only necessary to fit *b*_*1*_, *b*_*3*_, and *b*_*5*_ in the ODE model. Model fits to the data were compared using Akaike Information Criterion (AIC).

As a preliminary to the data fitting procedure, the model was run for 5 years prior to the start of the first month of available temperature data in October 1959 using the average monthly temperatures from October 1960 to September 1961 because we did not know initial starting values for numbers of pupae, relative to the numbers of fed female adults caught. The initial number of parous adults and pupae at time *t* = 0 was set to 100 and the number of nulliparous adults to 25, and the model was solved at monthly time steps for comparison with values from the bioassay catch data. We fitted the model to the data using maximum likelihood, assuming the data were Poisson distributed. For 80% of months, the variance to mean ratio for the daily catch data was less than 1.5, and was between 1.5 and 4.0 for 20%, indicating that, in most circumstances, the variance was approximately equal to the mean. For each set of parameters, we first estimated parameter values using the stochastic simulated annealing algorithm [50] and then used updated parameter estimates in a final fit using Nelder-Mead [51].

A penalty was incurred for parameter estimates of *a*_*1*_ greater than 0.04 or less than 0.01, ensuring baseline adult mortality was within biologically reasonable limits, by stopping the function before computing the likelihood and automatically assigning a high negative log likelihood value [45]. A penalty was also incurred for model fits for which, on average, there were fewer than 50 tsetse between January 1965 and December 1984 because, during that period, sampling teams consistently obtained their quota of 50 flies in an afternoon. Confidence intervals (95%) were calculated for fitted parameters using the Fisher information matrix.

A peer reviewer noted that these confidence intervals allow for no uncertainty in the fixed parameters. To explore the importance of this, we refitted the model using the upper and lower limits of the 95% confidence intervals of the fixed parameters *b*_*1*_, *b*_*3*_, and *b*_*5*_ of the function for the temperature dependence of pupal mortality. All analyses were done in R [41] and are available online, with all the data required to reproduce figures, at the following website: https://github.com/jenniesuz/tsetse_climate_change.

## Results

### Temperature increase at Rekomitjie

**Fig 1:**
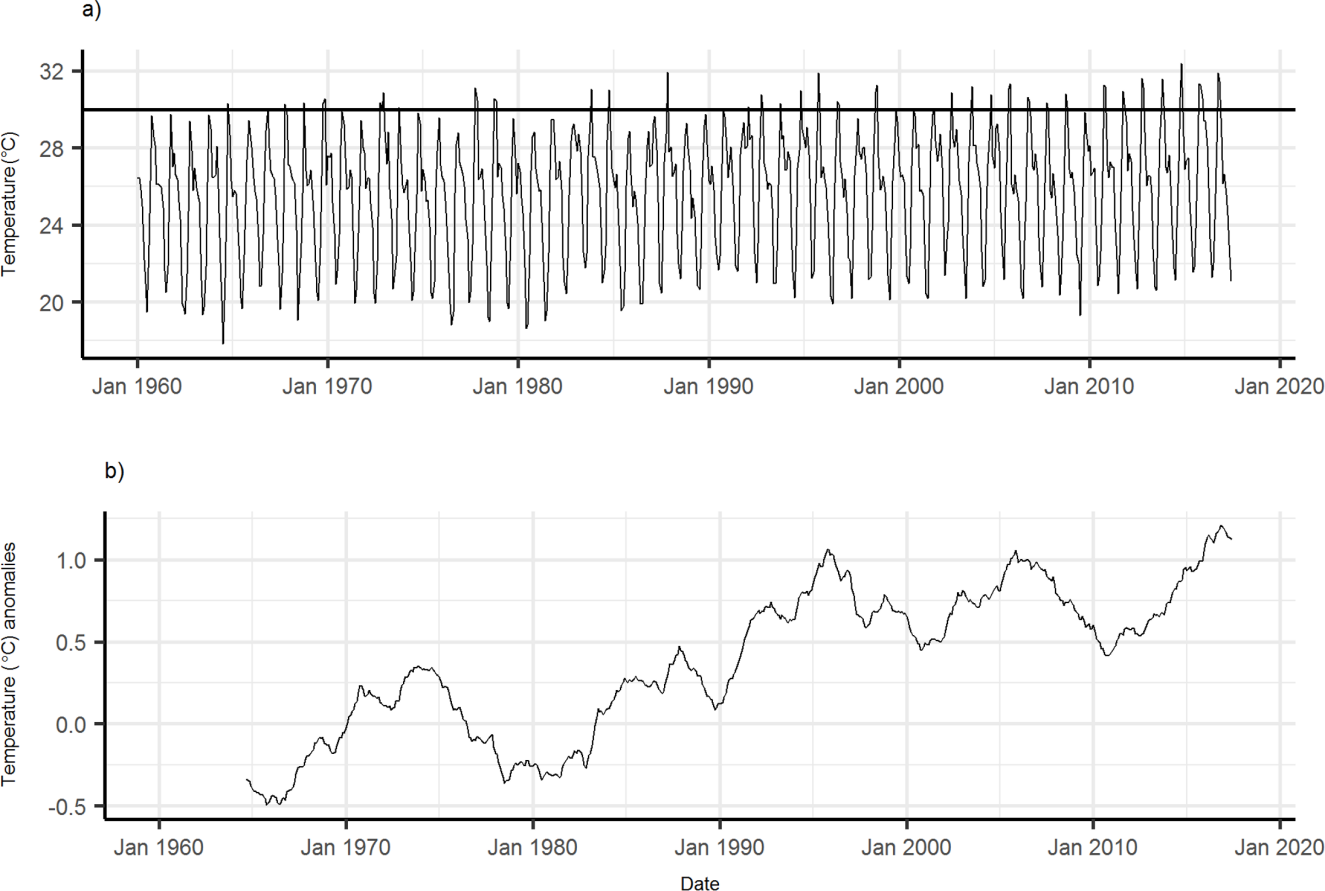
Temperature at Rekomitjie. (a) Monthly mean temperatures. Horizontal line at 30°C highlights the increase in the number of consecutive years during the hot-dry seasons in which mean monthly temperatures have exceeded this level. (b) Five-year running mean monthly temperature (°C) anomalies relative to 1960–1990 reference period.

**Fig 2:**
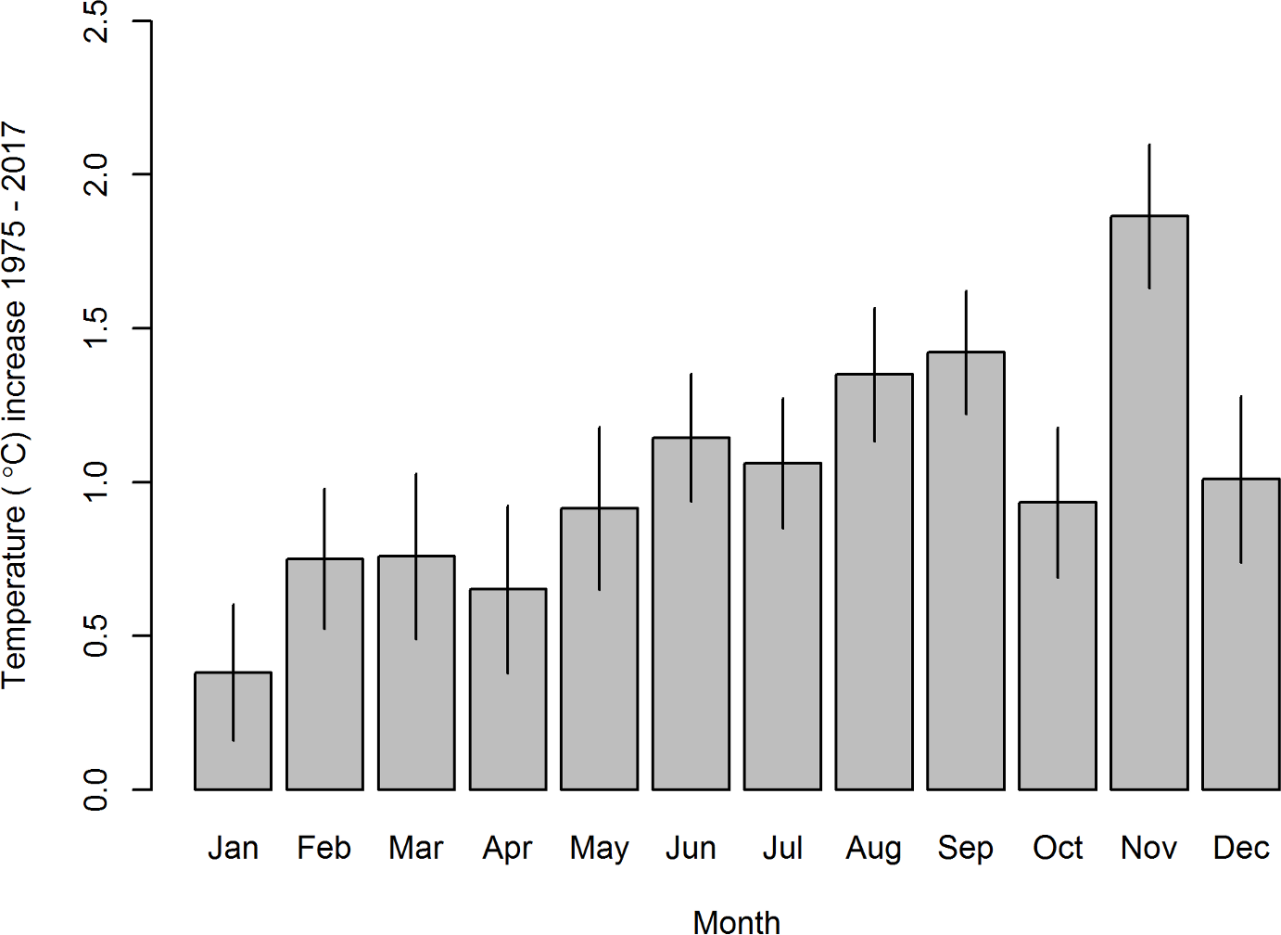
Increases in mean daily temperature between 1975 and 2017 calculated for each month of the year. Estimated using time series linear regression. Segments are 95% prediction intervals. All months except January and April had a statistically significant (*p* < 0.05) increasing trend between 1975 and 2017.

Although there is considerable seasonal and interdecadal variation in temperature (Fig 1A), our analyses indicate an increase of c. 0.9°C from the peak in 1975 to the peak in 2017 (Fig 1B). This increase is not even across the year, being greatest in November when temperatures are already highest. During this month, mean daily temperatures increased by c. 2°C between 1975 and 2017 (Fig 2). In addition, the number of consecutive years in which the hottest mean monthly temperature was above 30°C has increased since 1990 (Fig 1A).

### Modelling changes in the *G*. *pallidipes* population

**Fig 3:**
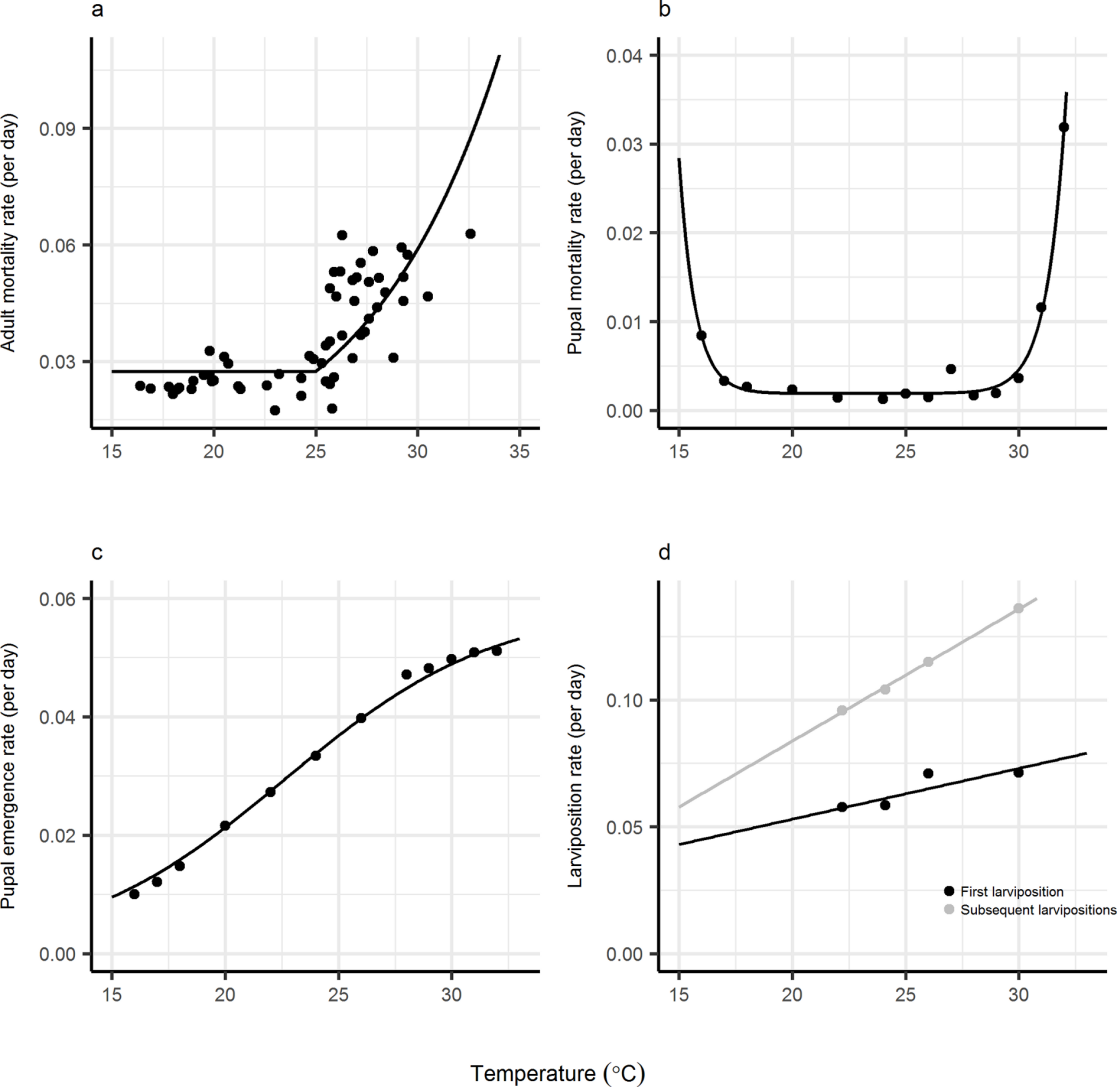
Fitted temperature-dependent functions. (a) Adult female mortality rate per day: points—published estimates from mark-recapture experiments on Antelope Island, Zimbabwe [30]; line—fitted temperature-dependent adult mortality function (Eq 1). (b) Pupal mortality rate per day: points—published estimates from laboratory experiments [27]; line—fitted temperature-dependent pupal mortality function (Eq 2). (c) Pupal emergence rate per day: points—published estimates from laboratory experiments; line—Eq 3 fitted as described in [26]. (d) Larviposition rate per day: points—data from published field experiments [28]; lines—Eq 4 fitted as described in [30]. See Table 1 for fitted parameter estimates of the mortality functions.

Tsetse flies are poikilotherms, and their development and mortality rates are dependent on temperature [26–30]. We used four temperature-dependent functions, with starting parameters estimated from fits to published data for pupal and adult mortality, larviposition, and pupal emergence rates (Fig 3, Table 1), in an ODE model of tsetse population dynamics (Eqs 5–7).

**Table 1:** Summary of model parameters. Fixed parameter values used, and estimates (95% confidence intervals) from population dynamic model with lowest AIC (Fig 4). Fixed parameters estimated from published laboratory or field data and fitted using nonlinear least-squares regression, fixed estimates using this method shown ± standard error (Fig 3).

Parameter	Function or parameter definition	Estimate from fit of Eqs 1–4 to published laboratory and field data	Estimate from fit of population dynamic model
*a*_*1*_	Eq 1: Adult mortality rate (*μ*_*A*_)	0.027 ± 0.001	0.03365 (0.03363–0.03368)
*a*_*2*_		0.153 ± 0.020	0.1168 (0.1166–0.1169)
*b*_*1*_	Eq 2: Pupal mortality rate (*μ*_*P*_)	0.0019 ± 0.0004	Fixed
*b*_*2*_		0.006 ± 0.001	Fixed
*b*_*3*_		1.481 ± 0.681	Fixed
*b*_*4*_		0.003 ± 0.001	Fixed
*b*_*5*_		1.211 ± 0.117	Fixed
*c*_*1*_	Eq 3: Pupal emergence rate (*β*)	0.05884 ± 0.00289 (24)	Fixed
*c*_*2*_		4.8829 ± 0.0993 (24)	Fixed
*c*_*3*_		−0.2159 ± 0.0050 (24)	Fixed
*d*_*1*_	Eq 4: Larviposition rate (*ρ*)	Nulliparous: 0.061 ± 0.002 Parous: 0.1046 ± 0.0004 (26)	Fixed
*d*_*2*_		Nulliparous: 0.002 ± 0.0009 Parous: 0.0052 ± 0.0001 (26)	Fixed
*δ*	Density-dependent mortality coefficient	NA	0.00002357 (0.00002349–0.00002364)

**Abbreviations:** AIC, Akaike Information Criterion; NA, not applicable.

**Fig 4:**
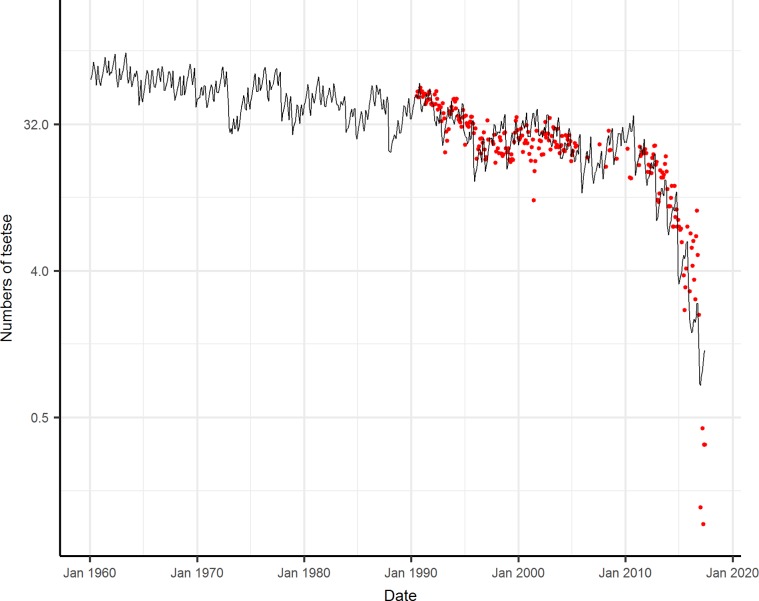
Observed (points) and modelled (line) changes in numbers of *G*. *pallidipes* females caught between 1960 and 2017. Data, on log base 2 scale, from 1990 to 2017, are average numbers caught by hand net, per afternoon, using an ox-bait. Fitted parameters are provided in Table 1.

The observed decline in catches of fed female *G*. *pallidipes* has continued to the present day, and the rate of decline has accelerated since 2010 to the point that teams now sometimes fail to catch a single fly in an afternoon (Fig 4). If these catches scale roughly with the population density of tsetse around Rekomitjie throughout the study period, the data suggest a steady decline in numbers over the last 27 years.

To simulate this decline, the model was run using mean monthly temperatures between October 1959 and June 2017. Model fits to the monthly tsetse catch data for 1990 to 2017 (Fig 4), varying *δ* and only the adult mortality parameters *a*_*1*_ and *a*_*2*_, while keeping all other parameters fixed, gave the lowest AIC of 1609 and provided a reasonable fit to the data (Fig 4). By comparison, varying only *δ*, or varying *δ* and parameters in Eq 2, or varying *δ* and parameters in both Eqs 1 and 2, produced AIC values of 2867, 1789, and 1764, respectively. Fixed and fitted parameter estimates for each function are summarised in Table 1. From 1959 until the mid-1980s, fitted model numbers of tsetse fluctuated between about 50 and 100 and then declined from c. 50 in 1990 to <1 in 2017, in good agreement with observed data. In addition, the fitted parameters *a*_*1*_ and *a*_*2*_ for adult mortality as a function of temperature (Eq 1) were similar to estimates from fits to the published mark-recapture data (S2 Fig, Table 1). The main difference was a higher baseline mortality for adults and a slower increase with temperatures above 25°C. When we carried out the sensitivity analysis, the upper and lower bounds for the coefficients for adult mortality were *a*_*1*_ = 0.024 and 0.030, and *a*_*2*_ = 0.145 and 0.198 (S1 Table).

Between 1959 and 2017, the pupal mortality rate was usually <0.005 in the fitted model. Prior to the 1990s, mortality was higher than this in October–December in 14 months over 30 years. In the 27 years since 1990, the pupal mortality rate was higher than this in 31 months during the hot-dry season. The hot-dry season is also the time of year when the modelled adult mortality was highest: >0.05 day^−1^. Adult and pupal mortalities in the fitted model were both above these levels in October and November more frequently in years after 1990. This is consistent with the idea that increasing temperatures during the hot-dry season are primarily responsible for the observed decline in numbers of tsetse at Rekomitjie since the 1990s, and particularly since 2000. Indeed, increases in mean daily temperatures have been most pronounced in November when temperatures are already highest (Fig 2).

The results of the preliminary analyses using constant values for larviposition and pupal emergence rate parameters are presented in S1 Text. The model with no temperature-dependent parameters did not provide a good fit to the data and had an AIC of 6762 compared to 2523 when adult temperature-dependent mortality was included (S1 Text).

## Discussion

While there are statistical models relating climate change to changes in vector populations [8,18–20], mechanistic models that relate climate change to data for the population dynamics of an important vector of human and animal pathogens are much less common. Our mechanistic model, incorporating the effects of temperature on mortality, larviposition, and emergence rates was sufficient to explain the observed decline in numbers of tsetse. The >99% decline in numbers reported here is comparable to the effects of successful large-scale tsetse control operations conducted in Zimbabwe.

Hargrove and Williams [52] found, similarly, that temperature was an indispensable factor in modelling tsetse population growth on Antelope Island, Lake Kariba, Zimbabwe. They had access to a wide range of measures of meteorological variables but found that once temperature had been included in their model, the addition of any further candidate variables—including rainfall, humidity, saturation deficit, and cloud cover—did not result in any improvement in the fit to the data. At Rekomitjie, over the whole study period, we had data only on temperature and rainfall. Nonetheless, the Antelope Island study suggests that we were unlikely to be missing other important climatological variables.

Our results provide evidence that locations such as the Zambezi Valley in Zimbabwe may soon be too hot to support populations of *G*. *pallidipes*. Similarly, *G*. *m*. *morsitans* populations at Rekomitjie are declining and might also be close to local extinction within the next few decades [53].

There are several biologically feasible reasons to expect increased tsetse mortality at high temperatures. Tsetse are poikilotherms, and their metabolic rate increases with temperature: adult tsetse therefore utilise their blood meal more rapidly at elevated temperature and must feed more frequently. But feeding is a high-risk activity, and increased feeding rates will likely result in increased mortality [54,55]. Tsetse use artificial refuge sites when ambient temperatures exceed 32°C [56], thereby reducing the temperatures they experience by up to 6°C during the hottest times of the day [57]. This behaviour reduces their metabolic rate but also reduces their chances of feeding. Hence or otherwise female tsetse have reduced fat levels and produce progressively smaller pupae as temperatures increase [58,59]. This has a knock-on effect on pupal mortality because smaller pupae can suffer very high mortality at elevated temperatures [47].

As temperatures increase, rates of pupal fat consumption increase linearly with temperature, whereas pupal duration decreases exponentially. The interplay between these rates results in fat levels at adult emergence being highest for pupae experiencing temperatures of about 27°C and progressively lower as temperatures increase above this level [26,47]. Reduced fat levels at adult emergence prejudice the chances of a teneral fly finding its first meal before fat reserves are exhausted and the fly starves or suffers excess mortality as a consequence of taking additional risks in attempting to feed [60]. The rate at which fat is used by teneral flies increases with temperature, exacerbating the above problems for the fly. There are also direct effects of high temperature on pupal mortality such that, when they are maintained at a constant level >32°C, no pupae emerge (Fig 3B) and all are found to have died before they utilised all of their fat reserves [47].

Few studies of vector-borne disease have been able to show a clear link between climate change and a change in either vector or pathogen population dynamics and subsequent disease burden [8]. Studies are frequently confounded by other environmental, ecological, and sociological factors, or the necessary empirical data are too difficult to collect. Although we acknowledge that this study presents only a first step in linking the effects of climate change to changes in the risk of acquiring a trypanosome infection, it suggests that climate change is already having effects on the density of disease vectors. In this respect, our study contributes vital analysis of long-term (>10 years) data in a region where temperatures have increased and where, as a consequence, the dynamics of a disease vector have also changed [8].

If these effects extend across the Zambezi Valley, then transmission of trypanosomes is likely to have been greatly reduced in this region. Conversely, rising temperatures may have made some higher, and hence cooler, parts of Zimbabwe more suitable for tsetse and led to the emergence of new disease foci. There is a pressing need to quantify the magnitude and spatial extent of these changes on tsetse and trypanosomiasis.

While there are no data on annual incidence of HAT from Zimbabwe to compare with the long-term data on tsetse populations, in the last 20 years, cases have been reported from the vicinity of Makuti [61], at the relatively high-altitude of c. 1,500 m, where tsetse populations are close to their low temperature limit. We are unaware of trypanosomiasis being reported from this area prior to the 1990s. This circumstantial evidence of the emergence of HAT in cooler regions of Zimbabwe raises the possibility of the resurgence of tsetse populations, and then of *T*. *brucei* infections, in parts of Southern Africa where they have been absent since the rinderpest epizootic of the late 1890s, apparently because the areas were too cold. Because tsetse dispersal is thought to arise through random movement [62], such a resurgence would come about where diffusion took tsetse to areas that are now climatically more suitable than they were in the recent past. Tsetse populations could only become established if, in addition, there were sufficient numbers of host animals and suitable vegetation to support tsetse. Hwange National Park in Zimbabwe and Kruger National Park in South Africa are examples of such areas, where suitable hosts and habitat for tsetse are abundant, and where tsetse did occur in the 19th century.

HAT is one of several vector-borne diseases for which detecting human cases is difficult even in countries with relatively strong health systems. In Uganda, for instance, it is estimated that for every reported case of Rhodesian HAT, another 12 go undetected [63]. In remote parts of the Democratic Republic of Congo (DRC), Central African Republic, and South Sudan, finding cases is even more difficult. In these circumstances, prospects for gathering data to detect or predict the impact of climate change on HAT seem poor. Models to predict where vectors are abundant, supported by xenomonitoring of tsetse populations for pathogenic trypanosomes [64], seem a more likely means of assessing the impact of climate change.

In general, if the temperature increase seen at Rekomitjie is reflected more broadly in the region, large areas that have hitherto been too cold for tsetse will become climatically more favourable and could support the flies if adequate hosts were available there [65].

In any region where the climate becomes more suitable for tsetse, there must, however, be adequate vegetation cover to provide shelter for the flies. The clearing of land for agricultural development, which is occurring at an accelerating pace in many parts of Africa [66], will reduce the vegetation cover and the densities of wild hosts in what has been termed the autonomous control of tsetse [67]. Any future predictions of the effects of climate change on tsetse populations and/or trypanosomiasis should consider these other confounding effects, as has been done for malaria [68]. Gething and colleagues [68] demonstrated that any future predicted changes in malaria due to climate would likely be magnitudes smaller than changes due to control and other anthropogenic factors.

Most (>95%) cases of HAT occur in Central and West Africa, where the important vectors are subspecies of *G*. *palpalis* and *G*. *fuscipes*, which are riverine tsetse. These species have very similar physiology to the savanna species of East and Southern Africa, including *G*. *pallidipes*, and hence we would expect that populations of riverine tsetse would decline if they were exposed to the temperature increases reported in the Zambezi Valley of Zimbabwe.

Over the past decade, c. 90% of all reported cases of Gambian HAT occurred in the DRC [69]. For the tsetse-infested regions of the DRC where HAT occurs (e.g., Provinces of Mai Ndombe, Kwilu, and Kasai), there are no data to suggest that climate change has had an impact on tsetse and HAT. For HAT foci in West Africa, however, there is some evidence that climate change has had an impact. First, Courtin and colleagues [70] describe a 100 km shift southwards in the northern limit of tsetse that they attribute to drought, rising temperatures, and increased human density. Regions where tsetse appear to be absent include areas in which sleeping sickness occurred in the 1930s. Second, Courtin and colleagues [71] report that, across West Africa, the more northerly foci of HAT located in Senegal, Mali, Burkina Faso, and Niger have ceased to be active. The authors attribute this change to increased densities of humans, anthropogenic destruction of tsetse habitat, and climate change. Medical surveys conducted between 2000 and 2006 did not detect any cases of HAT north of the 1,200 mm isohyet, and comparison of the 1,200 mm isohyet for the periods 1951–1969 and 1970–1989 show that it had shifted south.

For tsetse-infested areas of West Africa, Courtin and colleagues suggested that it is difficult to disentangle the effects that changes in land cover, host populations, rainfall, and temperature have on tsetse populations and sleeping sickness [70,71]. Studies are further confounded by the impact of large-scale medical interventions that have led to a decline in the annual incidence of Gambian HAT across Africa [72]. With such interpretive problems, there is a need for more studies of the present sort in which long-term measurements of tsetse abundance are made in wilderness areas where there is little change in land cover and host populations.

A limitation of our study is that the estimated confidence intervals for model-fitted parameters, such as those for adult mortality, are underestimates, in part because they incorporate only the uncertainty resulting from fitting the model with fixed values for other parameters and do not incorporate uncertainty in those fixed parameters. Another limitation is that we did not have sufficient data to test the predictive power of our fitted model.

Our deterministic model does, however, provide a good fit to available data for the change in tsetse abundance since 1990. Such models are less satisfactory for assessing if and when a population will actually go extinct because they predict that populations go to zero only as time goes to infinity. Ideally, therefore, future modelling should adopt stochastic approaches to predictions about tsetse extinction, but these would require detailed knowledge of population dynamics at very low density, such as the probability that male and female tsetse will meet in sparse populations. Unfortunately, our current knowledge of dynamics relates only to populations that are dense enough for convenient study. Nonetheless, present modelling does raise the possibility of the extinction of the Rekomitjie tsetse populations, particularly if temperatures increase further. Future research could also make use of the fitted model to make spatially explicit predictions about tsetse population dynamics for other regions of Zimbabwe and East and Southern Africa under future-predicted climate change scenarios.

## Supporting information

S1 Fig Study site location. Rekomitjie Research Station, located within Mana Pools National Park. Also showing woodland cover (2002) and loss (2000–2014) as estimated by Hansen and colleagues [23]. Source: Hansen/UMD/Google/USGS/NASA.
(TIF)

S2 Fig Adult temperature-dependent mortality. (TIF)

S1 Table Sensitivity analysis. Effect of varying pupal temperature-dependent mortality parameters on fitted parameter estimates.
(XLSX)

S1 Text Results of alternative model fits. (DOCX)

## Abbreviations

AAT: animal African trypanosomiasis
AIC: Akaike Information Criterion
DALY: Disability-Adjusted Life Year
DRC: Democratic Republic of Congo
HAT: human African trypanosomiasis
NA: not applicable
ODE: ordinary differential equation
