# Supplementary figures and images for: Climate change and African trypanosomiasis vector populations in Zimbabwe's Zambezi Valley: A mathematical modelling study
Source: PLoS Med. 2018 Oct 22;15(10):e1002675. doi: 10.1371/journal.pmed.1002675 (PMC6197628; doi:10.1371/journal.pmed.1002675)

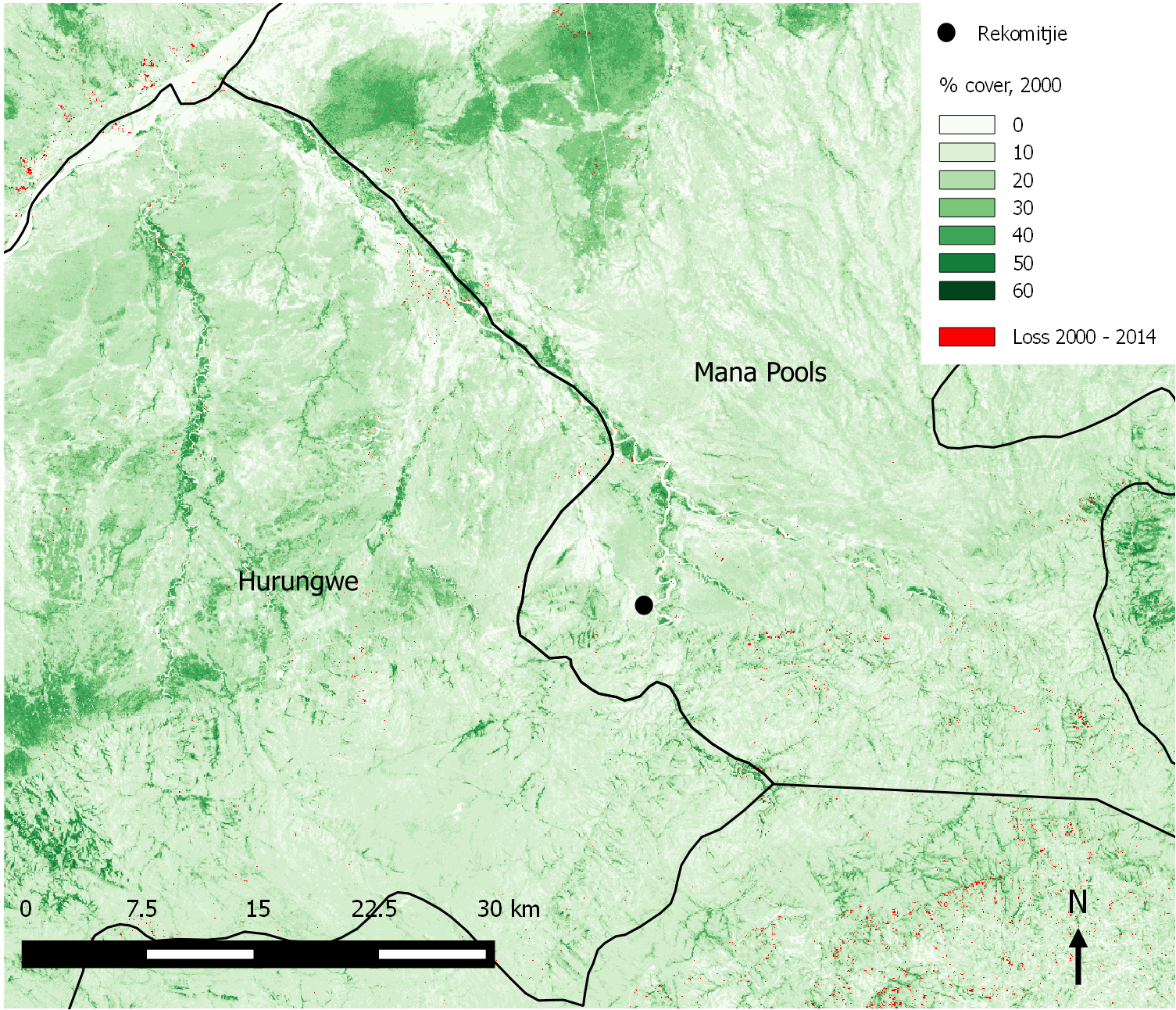

Supplement: S1 Fig — Rekomitjie Research Station, located within Mana Pools National Park. Also showing woodland cover (2002) and loss (2000–2014) as estimated by Hansen and colleagues [23]. Source: Hansen/UMD/Google/USGS/NASA. (TIF) [file pmed.1002675.s001.tif]

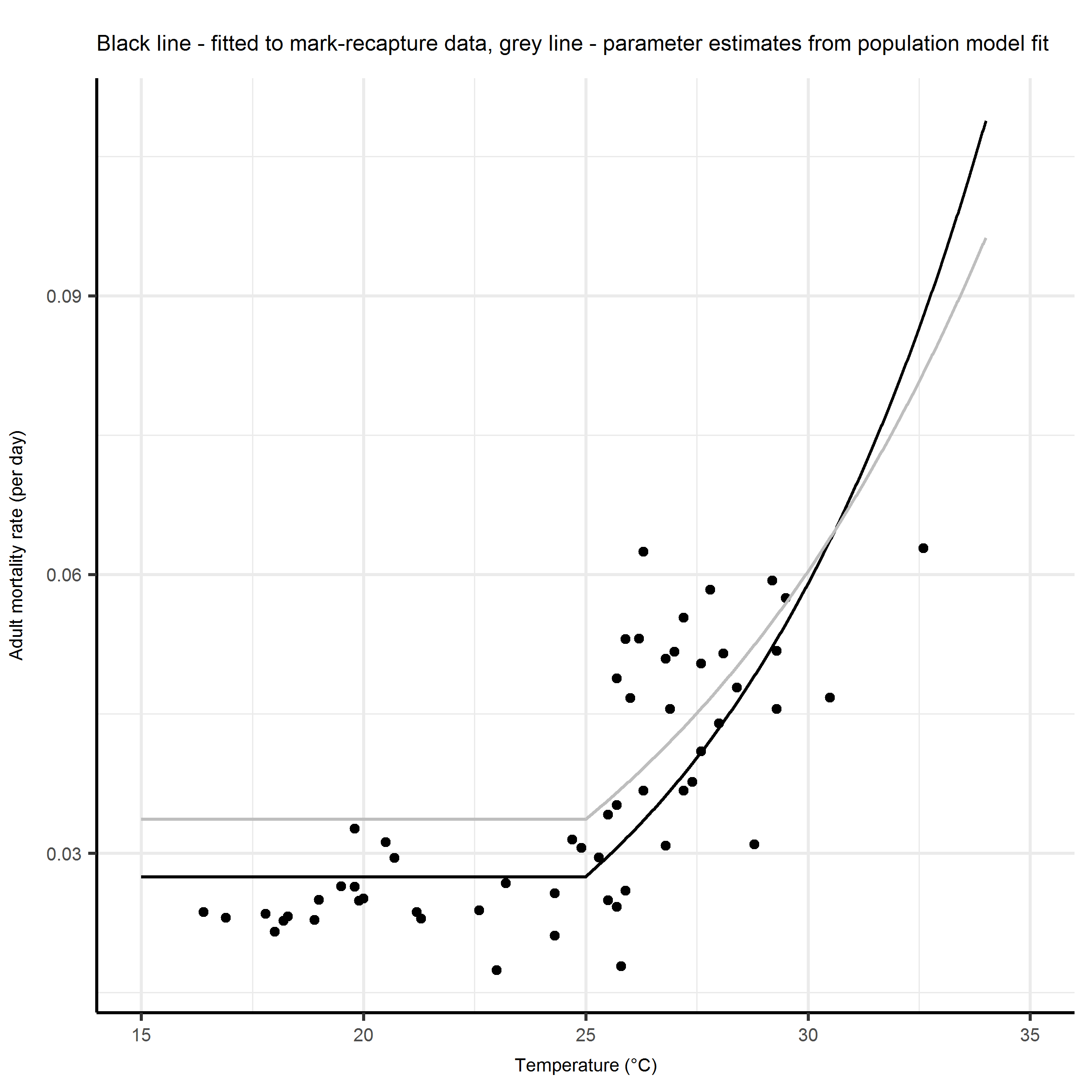

Supplement: S2 Fig — (TIF) [file pmed.1002675.s002.tif]
