# Supplementary material for: Climate change and African trypanosomiasis vector populations in Zimbabwe's Zambezi Valley: A mathematical modelling study
Source: PLoS Med. 2018 Oct 22;15(10):e1002675. doi: 10.1371/journal.pmed.1002675 (PMC6197628; doi:10.1371/journal.pmed.1002675)
Supplement: S1 Text — (DOCX) [file pmed.1002675.s004.docx]

**S1 Text. Results of alternative model fits**

Model 1: Constant larviposition, pupal emergence and pupal mortality rates, temperature-dependent adult mortality rate.

Model 2: Constant larviposition, pupal emergence, adult mortality and pupal mortality rates.

|  | **Fixed value** | **Fitted Value (95% confidence intervals)** | **AIC** |
| --- | --- | --- | --- |
| **Model 1** |  |  | 2523 |
| Pupal emergence rate (*β*) | 0.03/2* |  |  |
| Larviposition rate (*ρ*) | 0.1** |  |  |
| Pupal mortality rate (*µ_P_*) |  | 0.0021508 (0.002144 - 0.002158) |  |
| *a_1_**** |  | 0.0298 (0.0297 - 0.0299) |  |
| *a_2_* |  | 0.15780 (0.1573 - 0.1583) |  |
| Density-dependent mortality coefficient (*δ*) |  | 0.00000745 (0.00000742 - 0.00000747) |  |
| **Model 2** |  |  | 6762 |
| Pupal emergence rate (*β*) | 0.03/2* |  |  |
| Larviposition rate (*ρ*) | 0.1** |  |  |
| Adult mortality rate (*µ_A_*) |  | 0.0106134146 (0.01061340 - 0.01061343) |  |
| Pupal mortality rate (*µ_P_*) |  | 0.0031430251 (0.003143021 - 0.003143029) |  |
| Density-dependent mortality coefficient (*δ*) |  | 0.0004848568 (0.0004848562 - 0.0004848574) |  |

*Hargrove JW. Reproductive rates of tsetse flies in the field in Zimbabwe. *Physiol Entomol.* 1994;19(4):307–18.

**Phelps R, Burrows P. Prediction of the pupal duration of *Glossina morsitans orientalis* Vanderplank under field conditions. *J Appl Ecol.* 1969;6(2):323–37.

***See Equations in Methods section
